# Gut Dysfunction and Non-alcoholic Fatty Liver Disease

**DOI:** 10.3389/fendo.2019.00611

**Published:** 2019-09-06

**Authors:** Felix Grabherr, Christoph Grander, Maria Effenberger, Timon Erik Adolph, Herbert Tilg

**Affiliations:** Department of Internal Medicine I, Gastroenterology, Hepatology, Metabolism and Endocrinology, University Hospital Innsbruck, Innsbruck, Austria

**Keywords:** NAFLD, microbiota, inflammation, diabetes, liver

## Abstract

Non-alcoholic fatty liver disease (NAFLD) has emerged as one of the leading liver diseases worldwide. NAFLD is characterized by hepatic steatosis and may progress to an inflammatory condition termed non-alcoholic steatohepatitis (NASH), liver cirrhosis, and hepatocellular carcinoma. It became evident in the last years that NAFLD pathophysiology is complex and involves diverse immunological and metabolic pathways. An association between intestinal signals (e.g., derived from the gut microbiota) and the development of obesity and its metabolic consequences such as NAFLD are increasingly recognized. Pre-clinical studies have shown that germ-free mice are protected against obesity and hepatic steatosis. Several human studies from the past years have demonstrated that NAFLD contains a disease-specific gut microbiome signature. Controlled studies propose that certain bacteria with rather pro-inflammatory features such as Proteobacteria or *Escherichia coli* are dominantly present in these patients. In contrast, rather protective bacteria such as *Faecalibacterium prausnitzii* are decreased in NAFLD patients. Furthermore, various bacterial metabolites and microbiota-generated secondary bile acids are involved in NAFLD-associated metabolic dysfunction. Although these findings are exciting, research currently lack evidence that interference at the level of the gut microbiome is beneficial for these diseases. Further preclinical and clinical studies are needed to advance this aspect of NAFLD research and to support the notion that the intestinal microbiota is indeed of major relevance in this disorder.

## Introduction

Non-alcoholic fatty liver disease (NAFLD) has emerged in the last years as the most common liver disease worldwide (1). The reason for this development is mainly based on the observation that obesity and obesity-related disorders such as type 2 diabetes (T2D) are on the rise, and NAFLD reflects a typical complication of these disorders (2). Importantly, hepatic steatosis might evolve toward its inflammatory complication i.e., non-alcoholic steatohepatitis (NASH), liver cirrhosis, and hepatocellular carcinoma (3, 4). It is currently believed that ~10–20% of all subjects with NAFLD develop NASH. Whereas, advances in the NAFLD field have been substantial in the past years, it remains still unclear why certain subjects develop NASH while the majority of patients do not progress. Although it is clear that obesity plays a key role in NAFLD pathogenesis, aspects behind the obesity epidemic remain unresolved and might not simply reflect calorie consumption. It has long been assumed that certain host factors and particularly the gut microbiota is involved in the evolution of this disease toward its inflammatory phenotype i.e., NASH (5).

**Figure 1:**
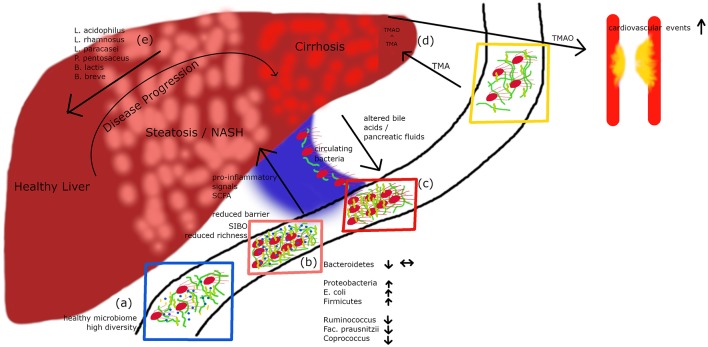
A healthy microbiome is characterized by high diversity of microbial species **(A)**. In patients with hepatic steatosis or NASH a small intestinal overgrowth develops and at the same time the diversity is decreasing **(B)**. Following impaired intestinal barrier function a rise in pro-inflammatory and pro-steatotic bacterial products in the portal circuit may occur, which can drive inflammation/steatosis and hepatic disease. Altered bile acids and pancreatic fluids can influence the microbiome. In cirrhotic patients, culturable bacteria can translocate via the portal vein to the systemic circulation **(C)**. Metabolites derived from the microbiome do not only influence the liver but also other organs. TMA is produced by bacteria out of dietary compounds, in the liver it is further metabolized to TMAO, which leads to an increased risk for cardiovascular events **(D)**. Recently published data (27) show that a cocktail of prebiotics can reduce the intrahepatic fat content in steatotic patients **(E)**.

The human gastrointestinal tract contains a huge number of bacteria, archaea, and viruses (6–10). The development of modern molecular and sequencing technologies allowed medical research in the last years to get some insights into this exciting bacterial world (11). Recent calculations proposed that three bacterial cells exist for every single cell of the human body (12). Whereas, it has been initially assumed that the genes of the gut microbiome mainly control functions regarding digestion of complex carbohydrates or regulating immune processes, recent evidence indicates that the microbiome has major functions in directing metabolic pathways in health and disease (13). The gut microbiota also controls the integrity of the intestinal epithelial barrier and thereby substantially contributes to gastrointestinal health (14–16) (Figure 1A). Indeed, evidence highlights that mucosal integrity is affected in obesity-related disorders including NAFLD (17).

It is increasingly acknowledged that the intestinal microbiota regulates metabolic functions and plays a role in the pathogenesis of NAFLD. Animal experiments suggested that the gut microbiota is critically involved in development of adipose tissue (18) and also affects evolution of hepatic steatosis (19). Indeed, early human studies found that obese subjects exhibit a gut microbiome signature (20). Several preclinical and clinical studies from the last years have investigated the role of the gut microbiota in fatty liver diseases and demonstrated that indeed a gut microbiome signature might be present in these disorders (21, 22). In addition, it seems likely that numerous bidirectional interactions exist between the liver and the gastrointestinal tract where the gut microbiota affects processes in the liver in health and disease and on the other side liver-released mediators such as bile acids control metabolic functions and the intestinal microbiota. We describe in this article recent evidence for a key role of the gut microbiome and related metabolites in the pathophysiology of NAFLD.

## Bacterial Dysbiosis in NAFLD

### Pre-clinical Studies

Landmark animal experiments performed by Bäckhed et al. suggested that the gut microbiota affects the development of adipose tissue (18). Equally important studies from Anna Mae Diehl's laboratory showed that the use of a certain probiotic decreased hepatic steatosis to a similar extent than treatment with an anti-TNF antibody (19) suggesting that both microbes and inflammatory pathways contribute to evolution of hepatic steatosis. One of the first convincing experimental studies supporting a key role for the gut microbiota in NAFLD came from a study by Le Roy and colleagues (23). In this study, the authors report that C57BL/6J mice developed similar obesity but responded metabolically differently toward a high fat diet (HFD): one group exhibited hyperglycemia and increased plasma concentrations of pro-inflammatory cytokines whereas the so-called “non-responder” group remained normoglycemic without significant systemic inflammation. In case germ-free mice were colonized with the gut microbiota from both groups, only mice receiving “responder” stool developed hepatic steatosis suggesting that intestinal microbiota allowed to transfer disease phenotype and liver steatosis (23).

Interference at the gut microbiome level by either pre-, pro-, or antibiotics might therefore affect disease phenotype. A prebiotic i.e., guar gum substantially changed the gut microbiota composition in a HFD model, decreased diet-induced obesity, and ameliorated glucose tolerance but interestingly even worsened liver phenotype leading to more inflammation and fibrosis (24). Chronic oral administration of an antibiotic in this model suppressed intestinal bacteria, reduced portal secondary bile acid levels, and decreased liver inflammation and fibrosis suggesting an important link between changes in intestinal microbiota, liver inflammation and fibrosis at least in a pre-clinical model probably via alterations in bile acids (24). Interestingly, manipulation at the level of intestinal microbiota might even improve more advanced NASH (25). In this study, rats were fed a HFD and high glucose/fructose syrup (HFGFD) and exhibited NASH including portal hypertension. Importantly, portal hypertension was improved after fecal transplantation from a healthy rat (25).

Obesity rates are increasing worldwide and maternal obesity confers risk to developing disease in offspring (26). In this recent study, the authors compared germ-free mice colonized with feces from 2-week-old infants born to obese or normal-weight mothers. Mice exposed to stool from infants born to obese mothers demonstrated enhanced liver gene expression for pathways such as endoplasmic reticulum stress and innate immunity combined with histological evidence of periportal inflammation. In addition, these mice exhibited increased intestinal permeability. When mice were challenged by Western-style diet, these mice showed excess weight gain and accelerated NAFLD. This study demonstrated an important interaction between motherhood obesity, infant dysbiosis and childhood obesity and NAFLD (26). From all these preclinical studies we can conclude that evidence for a role of the gut microbiota in NAFLD and related phenotypes is accumulating.

### Clinical Evidence for Dysbiosis in NAFLD

Substantial evidence from the past years suggests that the gut microbiota plays a role in NAFLD. In a clinical study including 22 subjects with NASH and 23 control patients, subjects with NASH showed small intestinal bacterial overgrowth (Figure 1B) and increased circulating endotoxin and TNFα levels (28). A meta-analysis of five clinical trials including 128 NAFLD patients and 83 control subjects revealed impaired intestinal permeability. These changes were even higher in the subgroup of NASH patients (29). A remarkable case report described a patient with small intestinal bacterial overgrowth, which evolved after jejuno-colic bypass surgery and was reversed after surgical correction (30). NASH patients exhibit a higher prevalence of SIBO and this has been linked with an increased expression of TLR4 on CD14 positive monocytes and higher plasma IL-8 levels (31). First reports assessing the gut microbiome in NAFLD patients were presented a few years ago (32, 33). In several studies, differences were abundant at phylum, family, and genus levels between healthy controls and NASH subjects (Table 1). Importantly, fewer differences in their microbiome were observed between obese and NASH subjects. *Proteobacteria, Enterobacteriaceae*, and *Escherichia* were the only phylum, family and genus types showing significant differences between obese and NASH patients (32). Another study demonstrated that patients with NASH had a lower rate of *Bacteroidetes* compared to steatosis and healthy controls (33). This data implicate that there exists an inverse association between the presence of NASH and *Bacteroidetes* content in feces. In a pediatric NAFLD study, *Actinobacteria* were increased whereas *Bacteroidetes* were reduced (34). Furthermore, at species levels *Oscillobacter* was lower in NAFLD whereas *Ruminococcus, Blautia*, and *Dorea* were increased in NASH (34). A further study assessed the gut microbiota and severity of histology-proven NAFLD in 57 patients (35). Here, *Bacteroides* abundance was enhanced and correlated with disease severity, whereas *Prevotella* abundance was decreased. In this study *Ruminococcus* abundance increased in more severe disease especially in advanced fibrosis. In conclusion, these studies underline that NASH is associated with a “microbiome signature” which could promote disease progression and the clinical phenotype.

**Table 1:** Overview of intestinal microbiota changes in NAFLD.

Enriched in NAFLD	*Bacteroides(Genus) (35)*
	*Ruminococcus(Genus) (35)*
	*Lactobacillaceae(Family) (36)*
	*Lactobacillus(Genus) (36)*
	*Proteobacteria(Phylum) (22)*
	*E. coli(Species) (22)*
Decreased in NAFLD	*Actinobacteria(Phylum) (34)*
	*Bacteroidetes(Phylum) (34)*
	*Oscillobacter(Genus) (34)*
	*Prevotella(Genus) (35)*
	*Ruminococcus(Genus) (36)*
	*Faecalibacterium prausnitzii(Species) (36)*
	*Coprococcus(Genus) (36)*
	*Firmicutes(Phylum) (22)*

Interestingly, Schierwagen et al. recently detected a circulating microbiome when studying central, hepatic and portal venous blood and peripheral blood from seven liver cirrhosis patients receiving a transjugular portosystemic shunt (37) (Figure 1C). Proteobacteria dominated in the circulation and changes were compartment-specific. Interestingly, in a small group of patients the investigators were able to cultivate respective bacteria suggesting that detected bacteria are viable and potentially bioactive. They also observed a direct correlation with cytokine levels arguing that systemic inflammation detected in liver cirrhosis patients might be associated with gut-derived bacteria. A circulatory systemic blood microbiome signature has also been described in a small NAFLD study assessing obese subjects with fibrosis (38), although the fecal microbiome was not studied. Severe alcoholic hepatitis is probably one of the diseases with the most severe impaired epithelial barrier. Another study found that these patients show a microbiome profile with a decrease in *Bacteroidetes* and an enrichment of *Fusobacteria*, bacteria which are mainly found in the oral cavity (39). This phenomenon was accompanied by higher endotoxemia and activation of type II secretion which has been related with the virulence of gram negative bacteria (39). Although these first studies open an exciting avenue, many more careful studies are needed to prove a relevance for “systemically” detectable microbiome.

The most convincing evidence for a potential role of the intestinal microbiota in human NAFLD has been reported by Loomba et al. (22). In this study, which assessed 86 patients with biopsy-proven NAFLD, the authors characterized 37 different bacteria which enabled to define mild vs. advanced fibrosis. Advanced fibrosis was characterized by an increased abundance of *Proteobacteria* and *E.coli* and a decrease in *Firmicutes* supporting the notion that a panel of microbiome markers potentially enables to diagnose advanced fibrosis in NAFLD. The severity of liver disease in NAFLD also might correlate with microbiome patterns (35). *Bacteroides* concentration was increased dependent on severity of disease, whereas *Prevotella* abundance decreased. *Ruminococcus* increased in more severe disease if advanced fibrosis was present (Table 1). Loomba's group recently reported a gut microbiome signature specific for NAFLD cirrhosis (21). In this study, they found that a panel of 30 features, including a panel of 27 bacteria, discriminated NAFLD-cirrhosis in a random forest classifier model. They strengthened their data by including both a derivation and validation cohort showing similar results (21). Some evidence has been reported that there is an association between a disturbed gut microbiome in NAFLD independent from the presence of obesity or insulin resistance (36). In this prospective cross-sectional study 39 adults with biopsy-proven NAFLD (15 simple steatosis, 24 NASH, and 28 healthy controls) were investigated. In NAFLD, *Lactobacillus* and Lactobacillaceae were more abundant compared to healthy controls. Lower abundance in both NAFLD patients compared to healthy controls were confirmed by qPCR for *Ruminococcus, Faecalibacterium prausnitzii*, and *Coprococcus*. Importantly, the lower abundance in NAFLD was independent of body weight and insulin resistance (36). Especially findings on Ruminococcus are rather controversial, as two other studies showed an increase in its presence in NAFLD suggesting that further studies are needed (34, 35). Only very few interventional studies in human NAFLD are so far available (27). This study assessed the effects of a probiotic treatment consisting of *Lactobacillus acidophilus, L. rhamnosus, L. paracasei, Pediococcus pentosaceus, Bifidobacterium lactis*, and *B. breve* on visceral and intrahepatic fat in NAFLD. Sixty-eight obese NAFLD patients were treated with either a probiotic or placebo for 12 weeks. Interestingly, body weight and total body fat decreased in the probiotic group but not in the placebo group. Treatment with this probiotic for 12 weeks decreased intrahepatic fat and body weight in obese NAFLD patients (Figure 1E) (27). Overall, evidence is evolving that there exists similar to obesity (40) and type 2 diabetes (41) a “gut microbiotal signature” in NAFLD which might allow us in the future to differentiate between patients with simply fatty liver and NASH and might furthermore allow to elucidate underlying pathomechanisms in the development of NASH. This also holds the promise that manipulation at this level might improve disease phenotype.

## What can we learn from microbiome studies in type 2 diabetes?

Several studies from the past years have suggested that a gut microbiome signature exists in diabetes (42, 43). Interestingly, in certain studies bacterial strains could be defined which are potentially involved in the regulation of certain features of T2D such as insulin resistance. Pedersen et al. found that the human intestinal microbiome affects the serum metabolome exhibiting increased levels of branched-chain amino acids (BCAA) and this was associated with insulin resistance in non-diabetic subjects (44). *Prevotella copri* and *Bacteroidis vulgatus, two* bacterial strains being identified in T2D subjects, caused insulin resistance in murines and upregulated levels of BCAA in these animals (44). Bacterial DNA was detected in mesenteric adipose tissue suggesting an impaired intestinal barrier and in another study in T2D patients *Ralstonia pickettii* was one of the most prevalent bacterial strains (45). The intestinal epithelium plays an important role in health and metabolic functions and an exciting recent study demonstrated that glucose is able to affect intestinal mucosal integrity (46). Certain bacterial strains such as *Eubacterium hallii* might be beneficial and are able to improve insulin resistance in rodent experiments (47). *E.hallii* treatment improved energy expenditure, enhanced fecal concentrations of butyrate, and affected bile acid metabolism. Similar to the Pedersen study (44), a recent report also observed that patients with pre-diabetes exhibit an aberrant intestinal microbiota accompanied by a decrease of *A.muciniphila* (48).

What comes first remains a key question. Hyperglycemia impairs epithelial integrity (46) and could exhibit a relevant factor influencing composition of the intestinal microbiota. In addition, an altered microbiota early in disease evolution of T2D could subsequently affect diverse disease characteristics of diabetes such as insulin resistance. Studies from pre-diabetes suggest that dysbiosis might reflect a rather early change in this disease (44, 48). Additional studies investigating well-characterized patients taking care of many potential additional factors such as dietary factors, drugs, or stool consistency are awaited to further strengthen the association of an altered intestinal microbiota with diabetes. The final proof, however, for a key role of the intestinal microbiota in type 2 diabetes depends on clinical trials where such new identified bacteria when administered to patients will demonstrate beneficial metabolic effects.

## Role of bacterial metabolites in NAFLD

The gut microbiota contributes substantially to circulating metabolites in the human body. These metabolites may act pro-inflammatory, direct metabolic processes, act protective, and anti-inflammatory or as probably true for most cases exert rather unknown biological functions. A recent chemical genetic screen revealed that many bacterial members of the gut microbiota release metabolites commonly interacting with so called G-protein coupled receptors (GPCRs) (49). This is potentially of major biological relevance as GPCRs are expressed by many cell types throughout the body and exert mainly immune and metabolic functions (50, 51).

Some metabolites, such as short chain fatty acids (SCFA) acetate, butyrate, or propionate can only be produced by bacterial fermentation, that is digestion of complex carbohydrates. Fecal SCFAs concentrations correlate with the presence of certain bacteria such as *Alistipes, Barnesiella, Prevotella*, or inversely with *Bacteroides* at least in mice (52). SCFAs are metabolically highly active, mainly act via GPCRs, such as Gpr41 with its ligand propionate (53). Importantly, Gpr41^−/−^ mice do not exhibit obesity despite high calorie challenge (54). The exact mechanisms of SCFAs in NAFLD are incompletely understood. Some evidence suggests that SCFAs promote gluconeogenesis and lipogenesis (55) which is also supported by clinical evidence (56). In contrast, many studies in the field of metabolism suggest that they act metabolically beneficial (57, 58). Butyrate is also suppressing HFD-induced obesity and insulin resistance (59). Another protective mechanism of SCFAs might be mediated via blocking histone deacetylases (HDACs) and thereby inducing regulatory T cells which could reduce insulin resistance (60–64).

Dietary factors such as phopsphatidylcholine, choline and carnitine are metabolized by the gut microbiota to generate trimethylamine (TMA) which is converted in the liver to TMA-N-oxide (TMAO) by flavin-containing monoxygenases. TMAO levels significantly relate with atherosclerosis and associated cardiovascular complications such as myocardial infarction and stroke as this metabolite promotes inflammation and platelet activation (65, 66) (Figure 1D). Increased TMAO levels correlate with the presence of T2D, glycemic control in T2D, its complications and also NAFLD (67–70).

A large study assessed the relationship between metabolome, the gut microbiome and hepatic transcriptome (71). Amount of hepatic steatosis was accompanied by a certain microbiome signature defined by decreased microbial gene richness (MGR), and increased rates of Proteobacteria, Actinobacteria, and Verrucomicrobia. A reduced MGR also correlated with an increase in branched chain amino acids (BCAA) pool and one bacteria-derived metabolite i.e., phenylacetate was highly associated with steatosis. Phenylacetate, a degradation product of certain essential amino acids such as phenylalanine and tyrosine and possibly other related metabolites enhance hepatic lipid accumulation by increasing BCAA utilization. Interestingly, administration of phenylacetate to mice resulted also in hepatic steatosis although underlying mechanisms are currently not understood.

Interestingly, some metabolites may interact with key metabolic pathways without affecting immune pathways or inflammation. Koh et al. recently identified such a metabolite by characterizing the bacterial metabolite imidazole propionate as a gut-derived factor regulating insulin signaling (72). The authors analyzed portal vein blood from patients with T2D for potential metabolically active factors. When the authors finally characterized imidazole propionate as a microbially produced histidine-derived metabolite they observed that subjects with T2D not only had higher circulating concentrations of this metabolite but also observed that imidazole propionate affected insulin signaling by activation of p38γ MAPK and phosphorylation of p62 which finally resulted in activation of mechanistic target of rapamycin (mTORC1). This study nicely illustrates how bacterial metabolites besides directing key inflammatory pathways might also control other key pathways of metabolic dysfunction (72). The here discussed metabolites reflect only some recently characterized microbiota-driven or microbiota-derived metabolites highlighting the enormous potential of this rather novel field.

## NAFLD: a prototypic systemic inflammatory disease

Low-grade inflammation is a hallmark of many chronic disorders such as chronic liver diseases or T2D and it remains currently unclear which compartments in the body might contribute to this phenomenon. Several NAFLD studies have shown increased circulating inflammatory parameters including cytokines, acute phase proteins, and adhesion molecules especially in NASH patients (73–76). Detectable systemic inflammation in these metabolic “non-communicable” disorders is usually considered sterile and driven mainly by innate immune factors (77, 78). Recent data from microbiome research, however, suggest that the gastrointestinal tract with its harbored microbiota might reflect an important source of inflammatory mediators and metabolites (5).

Low-grade chronic inflammation is of major relevance for NAFLD patients within and outside the liver. Although NAFLD patients most commonly die because of cardiovascular complications and atherosclerosis (79, 80), death due too liver disease is also frequent. Complications of NAFLD such as NASH, liver cirrhosis, and hepatocellular carcinoma are mainly but not solely inflammation-driven. It is now well-accepted that the amount of fibrosis defines the long-term prognosis of NAFLD-associated liver disease (81, 82), which is in most instances driven by inflammation. Therefore, inflammation substantially contributes to patient outcome both in (liver cirrhosis) and outside the liver (atherosclerosis, cardiovascular complications) in NAFLD.

Inflammatory events in case of NAFLD also have major metabolic consequences i.e., regulation of insulin signaling and insulin resistance. Indeed, insulin resistance has long been assumed to be controlled by inflammatory pathways (83). Many immune factors especially certain proinflammatory cytokines have been demonstrated to affect insulin signaling in various tissues. These studies are, however, dominated by preclinical reports and final prove in humans for such a role is still somewhat missing. Many studies have assessed the role of innate immune factors and cytokines in insulin signaling. We have recently shown, that interleukin 37 (IL-37), a human anti-inflammatory cytokine plays an adipokine-like anti-inflammatory and metabolically beneficial role in metabolic dysfunction (84, 85). Patients undergoing substantial weight loss consequent to bariatric surgery exhibited a massive increase of subcutaneous adipose tissue IL-37 expression similar as observed for adiponectin (84). When using transgenic IL-37 (IL-37tg) mice we observed substantial improvement of metabolic dysfunction in various models of obesity-related disorders including improvement of insulin sensitivity (85). Importantly, we also observed a steady-state IL-37 mRNA expression level in humans that directly correlated with insulin sensitivity and indirectly with degree of inflammation in adipose tissue. Type I interferon signaling induced by innate and adaptive immunity controls inflammatory processes subsequent to infection. The role of type I interferon signaling in metabolic diseases and the development of NAFLD is unclear. We recently assessed the role of type I interferon signaling in tissue-specific type I interferon receptor (IFNAR1) knockout mice (86). Adipose-, but not hepatic tissue-specific deletion of IFNAR1 worsened metabolic functions associated with a HFD characterized by enhanced weight gain, insulin resistance, and an impaired glucose tolerance compared to wild-type mice. Interestingly, deleted type I interferon signaling in myeloid or intestinal-epithelial cells did not modulate susceptibility to metabolic or hepatic disease. This study points out that type I interferon signaling in adipose tissue might play a role by in metabolic disorders such as NAFLD. A detailed discussion of this topic is beyond the scope of this article.

In conclusion, recent data indicates that chronic inflammation possibly derived from the gut microbiota substantially contributes to metabolic disorders such as NAFLD, which is a key event that guides patient outcome.

## Liver-derived signals affecting the gut microbiome: bile acids as key players

Whereas, life-long regulation of the composition of the intestinal microbiota is crucial for health and extrinsic factors such as dietary components reflect probably the key confounders, it is increasingly recognized that intrinsic factors with close proximity to the gastrointestinal tract such as bile or pancreatic fluid might be of equal importance (15, 87, 88). Bile acids not only regulate major metabolic pathways via interaction with mainly so-called nuclear receptors but also affect growth and regulation of the intestinal microbiota (15). Bile acids are generated by the liver from cholesterol and metabolized in the intestine by the intestinal microbiota. They signal via the nuclear farnesoid X receptor (FXR) and the G protein-coupled membrane receptor 5 (TGR5), which control various metabolic and immune pathways in the host.

The primary bile acids cholic acid and chenodeoxycholic acid in humans and muricholic acid in mice are produced in the liver as cholesterol derivates and secreted into the intestine as glycine and taurine conjugates. In the gut, these secondary bile acids interact with various nuclear receptors such as FXR, TGR5, pregnane X receptor, or vitamin D receptor (89). Recently it has been demonstrated that metformin affects the gut microbiota i.e., decreasing *B.fragilis* concentrations and in parallel resulted in an increase of the murine bile acid glycoursodeoxycholic acid (GUDCA) resulting in inhibition of intestinal FXR activation and respective metabolic benefits (90). Interestingly FXR activation in rodents was required for postoperative improvements in glycemia whereas changes in body weight, food intake, and other factors were unaffected (91). In this preclinical study bile diversion to the ileum improved glucose homeostasis via the FXR-Glp-1 axis and improvements were paralleled by an increase in intestinal *Akkermansia muciniphila* concentrations (91). We recently demonstrated that after massive weight loss after bariatric surgery (laparoscopic adjustable gastric banding, LAGB) both conjugated and secondary bile acids increased 3 months after LAGB and only one bile acid i.e., glycolithocholic acid sulfate remained significantly elevated even after 1 year (92). Furthermore, changes in bile acids importantly correlated with Glp-1 and fibroblast growth factor 19 levels supporting the notion that bile acid changes after LAGB crucially affect metabolic pathways. Many more studies have demonstrated in the past years that bile acids and derivates are crucial metabolic players and interference at the level of FXR could indeed reflect a promising treatment strategy for NAFLD in the future (93).

## Conclusions and Outlook

We discussed several pathways involved in prototypic gut-liver interactions. While a complex gastrointestinal bacterial world exerts and initiates biological processes throughout the body, underlying mechanisms are poorly understood. Studies from the past years have now convincingly shown that NAFLD patients at various stages of their liver disease have a specific gut microbiome signature. It becomes increasingly recognized that especially gram-negative bacteria such as Enterobacteria, *Escherichia coli*, and Proteobacteria are increased in concentrations and activities and may contribute to the pro-inflammatory phenotype of this disorder. One bacterial product which seems of key importance is endotoxin, which is elevated in advanced NAFLD (94, 95). It is still unclear which intestinal bacteria reflect the major source of this key driver of inflammation.

An equally exciting field is the topic of bacterial metabolites and their relevance in fatty liver diseases. Several excellent studies have proven that such metabolites regulate evolution of hepatic steatosis and insulin signaling. What is also increasingly acknowledged is the fact that gut-liver interactions take place bidirectionally and there exists also a major liver-gut axis meaning that products of the liver affect composition of the gut microbiome. Prototypic factors into this direction are mainly bile acids and especially secondary bile acids, which are again regulated by the gut microbiota. Bile acids and derivates interact and bind to nuclear receptors, which regulate key metabolic pathways involved in NAFLD. From all these studies it becomes evident that the interaction of gut bacteria, immunity and metabolic pathways are key to our understanding of this frequent liver disorder. Deciphering disease processes will allow to develop innovative treatment modalities that are desperately needed for NAFLD.

## Author Contributions

All authors listed have made a substantial, direct and intellectual contribution to the work, and approved it for publication.

### Conflict of Interest Statement

The authors declare that the research was conducted in the absence of any commercial or financial relationships that could be construed as a potential conflict of interest.
